# Characterization of simple sequence repeats (SSRs) from *Phlebotomus papatasi *(Diptera: Psychodidae) expressed sequence tags (ESTs)

**DOI:** 10.1186/1756-3305-4-189

**Published:** 2011-09-29

**Authors:** Omar Hamarsheh, Ahmad Amro

**Affiliations:** 1 Department of Biological Sciences, Faculty of Science and Technology, Al-Quds University, P.O. Box 51000, Jerusalem, Palestine; 2 Faculty of Pharmacy, Al-Quds University, P.O. Box 51000, Jerusalem, Palestine; 3 ANAHRI, Faculty of Medicine, Al-Quds University, Jerusalem, Palestine

## Abstract

**Background:**

*Phlebotomus papatasi *is a natural vector of *Leishmania major*, which causes cutaneous leishmaniasis in many countries. Simple sequence repeats (SSRs), or microsatellites, are common in eukaryotic genomes and are short, repeated nucleotide sequence elements arrayed in tandem and flanked by non-repetitive regions. The enrichment methods used previously for finding new microsatellite loci in sand flies remain laborious and time consuming; *in silico *mining, which includes retrieval and screening of microsatellites from large amounts of sequence data from sequence data bases using microsatellite search tools can yield many new candidate markers.

**Results:**

Simple sequence repeats (SSRs) were characterized in *P. papatasi *expressed sequence tags (ESTs) derived from a public database, National Center for Biotechnology Information (NCBI). A total of 42,784 sequences were mined, and 1,499 SSRs were identified with a frequency of 3.5% and an average density of 15.55 kb per SSR. Dinucleotide motifs were the most common SSRs, accounting for 67% followed by tri-, tetra-, and penta-nucleotide repeats, accounting for 31.1%, 1.5%, and 0.1%, respectively. The length of microsatellites varied from 5 to 16 repeats. Dinucleotide types; AG and CT have the highest frequency. Dinucleotide SSR-ESTs are relatively biased toward an excess of (AX)n repeats and a low GC base content. Forty primer pairs were designed based on motif lengths for further experimental validation.

**Conclusion:**

The first large-scale survey of SSRs derived from *P. papatasi *is presented; dinucleotide SSRs identified are more frequent than other types. EST data mining is an effective strategy to identify functional microsatellites in *P. papatasi*.

## Background

The sand fly *Phlebotomus (Phlebotomus) papatasi *(Scopoli) is a natural vector of *Leishmania major *(Yakimov & Schokov), which is the causative agent of zoonotic cutaneous leishmaniasis in the Middle East and other countries [1,2]. Simple sequence repeats (SSRs) or microsatellites, are common components of eukaryotic genomes and are short, repeated nucleotide sequence elements arrayed in tandem and flanked by non-repetitive regions [3,4]. SSRs often harbour high levels of polymorphism, in terms of repeat number, and have been developed into one of the most common classes of genetic markers due to their high degree of ubiquity, co-dominance and variability in number among individuals. In recent years, microsatellites were extensively used to investigate genetic variability and the population structures of a wide range of organisms, including parasites and vectors of infectious diseases [5-13]. In the absence of genome sequences for sand flies, the isolation of microsatellite markers was carried out using various enrichment methods [14,15]. This approach has led to the development of a panel of five polymorphic and informative microsatellite markers for *P. papatasi *[16-18].

Parallel to the rapid increase in availability of diverse DNA sequence data, which resulted from the huge advancement of sequencing techniques, labour-intensive methods for the generation of microsatellite markers have been replaced gradually by *in silico *data mining of genomic and expressed sequence tag (EST) datasets [19-21]. Microsatellites are effectively randomly distributed throughout the genome and can represent transcribed elements. Although, SSRs derived from transcribed ESTs can still maintain allelic variability comparable with that in non-transcribed genomic DNA, they can serve as molecular markers for numerous applications [22,23]. EST databases have been a rich source of SSRs for the development of "genotyping" applications. Marker development from already existing sequence data is rapid, efficient and economical. Any type of SSR will be detected using an appropriate search program, whereas only SSRs with pre-defined motifs are captured by enrichment. In addition, SSRs are physically linked to expressed genes and thus represent functional markers.

The aims of this study were to expand the genomic resources for *P. papatasi *by analyzing 42,784 ESTs available in the GenBank database, increase the number of SSR markers by mining a previously developed ESTs, and evaluate specifically designed primer pairs for their abundance and motif type.

## Results

### Sequence analysis

The sequence analysis of the whole data set comprised ESTs of an average size of 469 bp. Sequence composition showed slight bias toward A and T; A+T = 13,235,131 (56.5%), whereas G+C = 10,149,530 (44.3%). The frequency of the main nucleotides (A), (C), (G) and (T) were comparable: 28.7, 21.8, 21.5, and 27.8%, respectively.

### SSR types, distribution and frequency

Out of 42,784 ESTs analyzed; 1,499 (3.5%) SSRs were characterized. The number of repeats per SSR motif ranged from 5 to 16 repeats, with 5-9 being most frequent. On average, one SSR was found in every 15.55 kb of ESTs, and the total length of the regions containing repeats was 0.079% of the total ESTs size. A total of 93 ESTs were found to have more than one SSR motifs.

**Figure 1:**
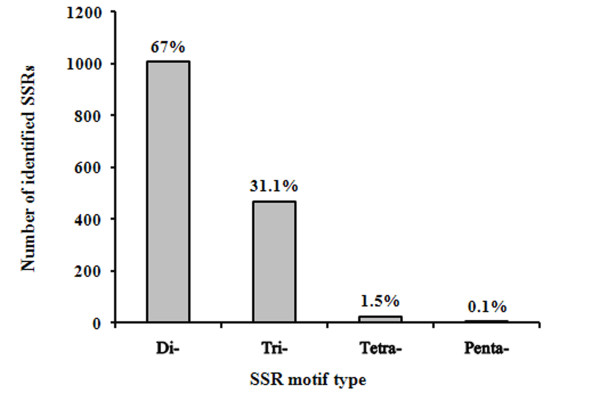
**Frequency distribution of different repeat types (2-5 motif units) identified in ESTs from *P. papatasi***. The numbers on the bars indicate the percentage of each repeat type microsatellites in total number.

**Figure 2:**
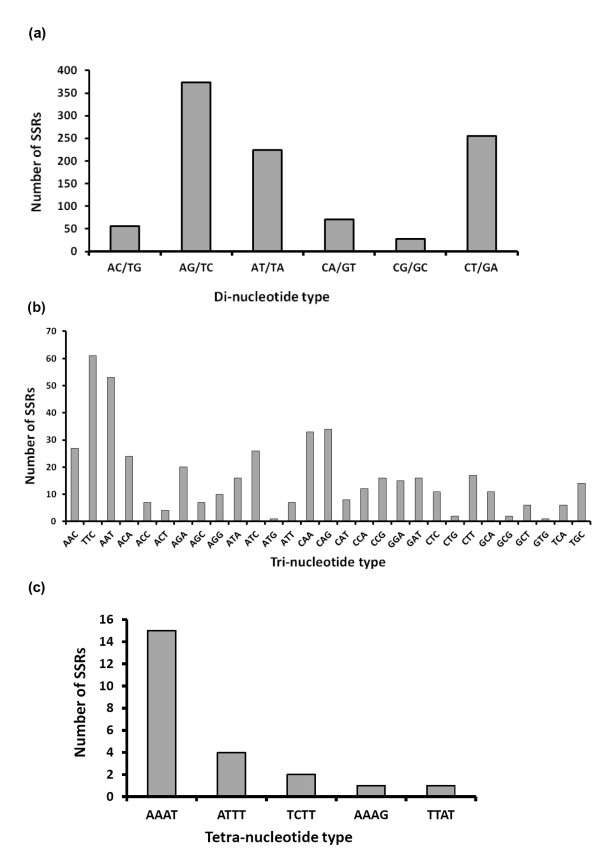
**Frequency distribution of (a) di-, (b) tri-, and (c) tetra-nucleotide repeat motifs of *P. papatasi***.

SSR loci were categorized by repeat type and structure; the dinucleotide repeat motifs were most abundant, accounting for 67% of the whole SSRs characterized followed by the trinucleotides (31.1%), tetranucleotides (1.5%), and pentanucleotides (0.1%), (Table 1). No hexanucleotide SSRs were detected in *P. papatasi *ESTs. Among the dinucleotide motifs, AG/TC type was more abundant (37%) than CT/GA (25.3%) and AT/TA types (22.2%); few CA/GT (7.1%), AC/TG (5.6%) and CG/GC (2.8%) types were characterized (Figure 1). For the trinucleotide SSRs, 467 motifs and 29 motif types were identified for *P. papatasi*; the TTC motif was the most abundant (13%), followed by AAT (11%), CAG, CAA (7%) each, AAC, ATC (6%) each, and ACA (5%) while the other motifs were at lower frequencies (Figure 2). Five types of tetranucleotide motifs were characterized; AAAT, ATTT, TCTT, AAAG, and TTAT, with frequencies of 15, 4, 2, 1, and 1%, respectively. Two identical pentanucleotide motifs of AATTG type (0.1%) were also identified.

**Table 1:** Summary of *in silico *mining of EST sequences for *P.papatasi *sand fly

Parameters	Number (%)
Total EST sequences	42,784
Total number of SSRs identified	1,499 (3.5%)
Frequency of SSRs	One every 15.55 kb

Numbers in parentheses is the percentage value of the repeat type.

### SSR marker development

Of 1,499 unique ESTs containing SSRs, 630 (42%) were suitable for primer design, comprising 425 dinucleotide, 271 trinucleotide and 9 tetranucleotide SSRs. The remaining sequences were inappropriate for primer design, mainly because of insufficient DNA sequence flanking the microsatellite core, or the sequences themselves not being suitable for primer design. Thus, overall SSR primers could be designed to amplify non-redundant loci from ~ 1.5% of the initial number of ESTs. Based on the size of repetitive motifs, a subset of 40 primer pairs were selected and designated as prime candidates to carry out polymorphism analysis using a minimum repeat length criterion of 5 repeats. This subset comprised 27 dinucleotide, 8 trinucleotide and 5 tetranucleotide (Table 2).

**Table 2:** Primers designed and suggested to amplify repeat sequence (SSRs) including number of repeats product size in bp, forward (Fw-) and reverse (Rv-) primer sequences, and melting temperature (Tm).

Name	**Accession no**.	SSR	Product size	Fw-Primer (5'-3')	Tm (°C)	Rv-Primer (5'-3')	Tm (°C)
PPEST1	EY218895.1	(CA)15	170	AGTTCCGCCACATCCATTC	60.9	TTAGACAGCGGGAAAGAAGAAA	60.4
PPEST 2	FG108562.1	(GCA)13	141	TGTCAATAGTGGCTCAATGCTC	60.3	ATTAGTCGTTTATCCTTCCCCG	60.5
PPEST 3	EX474573.1	(TA)13	188	CAATTTTATGCGGTCTATGGGA	61.0	AGGTATGCAAAGTAATGGGTGG	60.1
PPEST 4	FG116618.1	(TC)13	197	ACCCGACGCGAATTTACTTT	60.8	GGAGAGACAAGTTATGGGGTCA	60.4
PPEST 5	FG107376.1	(TGC)13	190	GAGAGACATGGTGGATGGACTT	60.4	TGTCAATAGTGGCTCAATGCTC	60.3
PPEST 6	FG108078.1	(AT)12	291	AAATCCACTATCCTCCTTTCCTC	59.0	TTTTGGGGTAAGATGGGG	58.2
PPEST 7	EX473561.1	(CA)12	245	GTACCCTTTCCCTCCCTATGTC	60.1	GGGTTCACCAACATCCTCC	60.2
PPEST 8	FG119248.1	(CA)12	140	CCACTGTAACTTGAGGAGGAGG	60.2	AGACTTGATGAGTGCGTCTCTG	59.7
PPEST 9	FG117610.1	(CA)12	175	CGCACAAGAACAAAGTGGAAA	61.2	TCTTCTCGCTCCCTCGTTC	60.6
PPEST 10	FG117371.1	(TC)12	236	ACTGAATCTTCTGCTTTCTCCATTC	61.4	TAAGGGAAGGGGCGGAAC	62.2
PPEST 11	ES347986.1	(GA)11	162	GGTGGATACTTGTGACGACTGA	60.0	CCACTCAAACTAAACTGGAAAGC	59.4
PPEST 12	FG113351.1	(AT)10	233	CTTTTCTGCCTTAGCTGCGTT	61.0	CGTGTCTCTTCCACCACTACAA	60.2
PPEST 13	EY206382.1	(TC)10	222	AGCTGGAATCAGGAGCAAAT	58.9	CAGTATCAAGCGAAAGCCG	59.6
PPEST 14	FK815057.1	(GA)9	228	ACGTGTTGTTTTCTGTGGAGTG	60.1	CTGGGTATTTTCTGCCTTGATT	59.5
PPEST 15	FK812013.1	(TGA)9	157	AAGAAAGGTTTGGCTTCGTGT	60.2	AATGGTGCTTCATCTCCTCTTC	59.7
PPEST 16	FK811085.1	(TCA)9	239	TTCTGTTCACACATCATTTCCC	59.8	TGTGGCTGTAATTTGACTGGAG	60.2
PPEST 17	FG116712.1	(TGC)9	208	CTGTTCAGCAAAACGAGACG	59.6	TCCCAAGTACAAAGACGGAACT	60.0
PPEST 18	EY216123.1	(CT)9	276	ACTTGCATACTCTTTCGCACAA	59.9	AAATTCATGGAAAACCTCCCTC	60.5
PPEST 19	EY210796.1	(AC)9	211	ACCCATCACCGTCTCTGC	59.6	TTTCCCTTGAACAACAACCAC	59.9
PPEST 20	EY210288.1	(TC)9	293	ACAGAAGAAACCATCCATTTGC	60.4	CCATATTCCCGATTGAGAGAGA	60.4
PPEST 21	EX474074.1	(AG)9	244	GGATTAGTGTGGCTCAAGATGG	60.9	GCAGGAAAATAGCAAAAGGGAT	60.8
PPEST 22	ES347170.1	(GT)9	229	ATGGGGTATTAAGGGAGAATGC	60.4	GGGACGTGTGTGAGTGAGATAG	59.6
PPEST 23	FG120710.1	(GA)8	223	CTGCATTTCTAATTTCGCGG	60.7	GGAGGAAGTGGACAGTGAAAAC	60.0
PPEST 24	FG118096.1	(TC)8	129	AGGCACATTTTGGTTGTCTTCT	60.0	ATTTAGGGAGTCAATAGCGCAG	59.8
PPEST 25	EY218678.1	(TTTA)8	132	CCATTCACTTCAAATCCATCCT	60.2	AACTGGGTGGTTGGTTGTTTT	60.5
PPEST 26	EY216313.1	(AC)8	217	GATTCCCCAGGCAAAATAAA	58.9	TAATCAATATGGTGGGTTCCG	59.5
PPEST 27	EY213284.1	(TAAA)8	150	TTGCTAAAGACAAGCGCAACT	60.2	CCATTCACTTCAAATCCATCCT	60.2
PPEST 28	EY209870.1	(CAA)8	245	GATCAAGGCGGTTAATTTCAAG	60.0	ACAATCCAGAAGGACGATGC	60.1
PPEST 29	FK814636.1	(GGT)6	228	TTTGTGGAGTTCGATGACTACG	60.2	GGACACATTCCTGTTCCAATTC	60.6
PPEST 30	EY218675.1	(CT)9	373	TTCGCTCTTTCTCTCTCTCTCC	59.5	ATTCTGTACGTTACCTGCCCTG	60.4
PPEST 31	EY215872.1	(TA)11	400	AAACGTGCATTCTCTGCCTAAT	60.2	CTCGATATTTATTTCCCCGCT	59.5
PPEST 32	EY210648.1	(TTTA)6	123	ATTCACTTCAAACCCCTCCTTT	60.2	AACTGGGTGGTTGGTTGTTTT	60.5
PPEST 33	FG115100.1	(GAA)15	251	ATACTCCCTCAGAACTAGCCCC	60.0	TTCGTCTTCTTCTTCTTCCTCC	59.1
PPEST 34	EY215687.1	(AAAG)5	137	CACCTACAGAGATGCTGGATTG	59.8	GGGCTAAAATGTGTCTTGACTTG	60.0
PPEST 35	EY214242.1	(GT)15	245	TAGTCACAACACACGAACCACA	60.1	TTAACCGTGAGAGTACCAGCAA	59.8
PPEST 36	EY203279.1	(TTTA)6	123	ATTCACTTCAAACCCCTCCTTT	60.2	AACTGGGTGGTTGGTTGTTTT	60.5
PPEST 37	EX474189.1	(AT)7	208	ACCGTGCAACCATTTTAAGTTC	60.3	AGTTATTCTTCTTCTTACTGCGCC	59.5
PPEST 38	FK811878.1	(AG)5	256	TCCAGATACTCAAGTTCCAGCC	60.6	TATAGCGTTCAGATCCACCAGA	59.7
PPEST 39	FG107375.1	(CT)6	292	CCCCAAAGAGAGTACACCAAAG	60.0	ATCAGCCAGTGTCGTATGAATG	60.0
PPEST 40	FG114532.1	(AG)5	322	TCCCAAGGCTATTAAGTCTGGT	59.2	GGCTATCGTGCAATTTTCTTCT	59.8

## Discussion

Molecular markers are central for investigating genetic variability and for understanding genome dynamics. In the case of sand flies, the development of molecular markers, however, has remained slow. Microsatellites or SSRs have proven to be useful markers in population genetic studies of sand flies [16]. The presence of SSRs in coding regions suggests their importance as functional markers. While the development of microsatellite markers for sand flies from genomic libraries has been relatively costly, labour intensive and time consuming [14-16,18], the mining of microsatellite markers from EST data overcomes these disadvantages.

The ESTs used in the present study were normalized. Hence, redundancy in the EST database was minimized and a wealth of unique cDNA sequences (unigenes) for marker development was found. Examining the distribution of SSR motifs can assist in gaining insights into genome composition and genetic makeup [24,25]. Although, SSR motifs with more than five repeats were considered here, shorter SSRs were identified. The maximum length achieved was 16 repeats; this is consistent with studies that revealed shorter SSRs in *Drosophila *[26,27].

Dimeric repeat motifs were more abundant than trimeric repeats. However, this observation was expected, as the frequency and distribution of SSRs depend on several factors, such as size of dataset, and tools and criteria used for SSR discovery. Tetra- and penta-repeat motifs were considerably less represented.

The most abundant SSRs were of dinucleotide type (Figures 1 and 2), in which homopurine-homopyrimidine stretches, such as AG and CT, have the highest frequency. Dinucleotide repeats are typically more frequent in noncoding regions [28-30]; however, they occur occasionally in coding regions as well [31]. Some dinucleotides, such as (AG)n/(CT)n, are not selectively neutral and may have functional roles. These repetitive sequences occur in the 5'-UTR and are likely to be involved in gene regulation [32-34]. The (GC)n repeats were absent from *P. papatasi*, even though they are numerically abundant SSR loci in most eukaryotes [35,36]. However, dinucleotide SSR motifs in *P. papatasi *ESTs are relatively biased toward an excess of (AX)n repeats and a low GC base content, the broader implications of this observation are unclear.

The high frequency of dinucleotide motifs (AT, AG, and CT) could be explained by their abundance in several codons with different nucleotide arrangements. This observation is in agreement with previous reports [32,37]. EST-derived SSRs; AG/CT repeats have been studied widely in eukaryotes, particularly in plants, and found to be highly abundant and highly polymorphic [38,39]. For *P. papatasi*, the number of published SSR markers is very limited compared with other major insect vectors, including species of *Anopheles *and *Aedes *[14]. In the present study, an in-depth analysis of microsatellites, in terms of density, resulted in the development of a new set of 40 SSR markers (Table 2). Thus, we have shown that the mining of ESTs is an effective strategy to identify functional microsatellites, with perfect repeats, in *P. papatasi*.

The prevalence of trinucleotide SSRs in *P. papatasi *ESTs was expected, since they do not interrupt triplet codons, whereas other repetitive stretches, such as mono-, di-, or tetra-nucleotides lead to frame-shift mutations, which would result in severe adverse effects in coding regions. However, the abundance of trinucleotide SSRs in coding regions of various organisms was much higher than in non-coding regions of the genome [37,40-45]. In contrast, the present study showed that trinucleotides were the second most abundant SSRs in *P. papatasi *ESTs (31.1%) compared with dinucleotides (67%). This observation could be explained by the SSR mining tool used here and its preset criteria, such as identification of a minimum number of repeats, which could have led to the identification of more repeats. This approach could have led to the identification of more dinucleotides and fewer tri- and tetra-nucleotides, with this bias contributing to the over-representation of dinucleotides compared with tri- and tetra-nucleotides. Another possible explanation is that *P. papatasi *EST data do not contain many trinucleotide SSRs compared with dinucleotides.

## Conclusions

This is the first large-scale survey of 1,499 unique EST-SSRs of *P. papatasi*. Despite the number of EST sequences surveyed, SSR loci do not appear to be particularly dense or frequent in *P. papatasi *(3.5%). SSR repeats characterized are mainly of dinucleotide type and heterogeneously distributed across all potential base compositions, with a small number of GC-rich repeat motifs. The DNA replication machinery likely contributes to the elevated abundance of dinucleotide AT-, and AG- rich repeat motifs and to lesser extent trinucleotide motifs, suggesting that future screens of *P. papatasi *and other sand fly molecular markers may benefit by focusing on SSR motifs. The utility of the microsatellite markers characterized in this study should be evaluated in the near future. More microsatellite markers should be characterized for *P. papatasi *and other key sand flies of major importance as vectors of *Leishmania*.

## Methods

### Retrieval of EST sequences

*P. papatasi *EST sequences used were directly retrieved from NCBI database http://www.ncbi.nlm.nih.gov/projects/dbEST/ on May 10, 2011. A total of 42,784 *P. papatasi *ESTs were listed and annotated. These ESTs were derived from three cDNA libraries constructed from uninfected sugar fed, uninfected blood fed, and *L. major *infected blood fed *P. papatasi *sand flies. All the sequences were saved in FASTA-formatted text files that were used for further analysis.

### Characterization of SSRs

PolyA and polyT tracts were removed, leaving no (T)_10 _or (A)_10 _in any 10 bp window at either end of the sequences. The dataset was divided into small files, each containing 100 FASTA formatted sequences. SSR-containing sequences were identified using SSRIT web based SSR identification tool [46] available at http://www.gramene.org/db/markers/ssrtool. Any sequence was considered as an SSR where a repeat motif of one to six nucleotides in length was repeated at least five times for dinucleotide, trinucleotide, tetranucleotide and pentanucleotide SSRs. Redundant sequences were filtered by BLAST analysis, using each individual sequence as a query against the total set of selected sequences. Homologous sequences were aligned using MEGA 5 and scanned manually in the sequence editor window [47]. The criteria for redundancy were: (i) where a cluster contained two or more identical sequences, the longest was retained; (ii) sequences which were composed entirely of SSR motif, lacking any flanking sequence, were discarded since their uniqueness could not be established and in any event, primer design was not possible.

### Sequence analysis

Total number of characters, sequence composition frequency and A+T and G+C contents were carried out by CLC Genomics Workbench program, v.3.7 (CLC bio, Denmark). The EST sequences were screened for the presence of perfect SSRs, and repeat motifs ≥ 5, these sequences were selected, annotated and filed for primer design.

### Primer design

The non-redundant EST-SSRs were used for primer design to flanking sequences using PRIMER3 [48]. PRIMER3 was calibrated to the following parameters: (i) Primer length from 18-27 bases, the optimal annealing temperature (Tm) from 55 to 60°C, the target amplicon size 100-300 bp, and GC content between 30 and 70% (50% as the optimum). All other parameters were set to default values. The output from PRIMER3 was further analyzed in order to lessen the chance of encompassing tandem repeats in primer sequences and self- and pair-complementarity.

## Competing interests

The authors declare that they have no competing interests.

## Authors' contributions

OMH designed the study, conducted data analysis and drafted the manuscript. AHA participated in data analysis and drafting the manuscript. Both authors approved the final version of the manuscript.
